# Applications of Speckle Tracking Echocardiography in Stress Echocardiography: A Systematic Review on Feasibility, Diagnostic, and Clinical Utility

**DOI:** 10.1111/echo.70251

**Published:** 2025-08-06

**Authors:** Johannes Kersten, Achim Jerg, Johannes Kirsten, Hasema Persch, Sarah Krieg, Yuefei Liu, Jon Magne Letnes, Jana Schellenberg

**Affiliations:** ^1^ University Hospital of Ulm Ulm Germany; ^2^ Herzplus Ulm Ulm Germany; ^3^ Department of Circulation and Medical Imaging Norwegian University of Science and Technology Trondheim Norway; ^4^ Clinic of Cardiology St. Olavs University Hospital Trondheim Norway

**Keywords:** global longitudinal strain, myocardial strain, speckle‐tracking echocardiography, stress echocardiography

## Abstract

**Purpose:**

This review evaluates the applications of speckle‐tracking echocardiography (STE) in stress echocardiography, focusing on feasibility, physiological adaptations, and its diagnostic and prognostic utility in cardiovascular conditions.

**Methods:**

A systematic search of PubMed identified studies published up to December 28, 2024, using terms related to STE, stress echocardiography, and myocardial strain. Grey literature was excluded. Eligible studies assessed myocardial strain under stress conditions and reported on feasibility, diagnostic accuracy, or prognostic outcomes.

**Results:**

Sixty‐four studies were included, covering healthy individuals, athletes, at‐risk populations, and patients with cardiovascular conditions. Feasibility studies demonstrated high interobserver reliability, with strain parameters measurable in > 80% of cases (intraclass correlation coefficient [ICC] 0.80 to 0.92). In healthy individuals and athletes, STE revealed myocardial adaptations and exercise‐induced changes. In at‐risk populations, such as those with diabetes or after cardiotoxic therapies, STE identified subclinical myocardial dysfunction, facilitating early intervention. For coronary artery disease (CAD) and heart failure (HF), stress‐induced changes in global and segmental strain improved diagnostic accuracy and risk stratification, with thresholds such as a > 15% reduction in global longitudinal strain under stress aiding therapeutic decision‐making.

**Conclusions:**

STE enhances the diagnostic and prognostic utility of stress echocardiography by providing reproducible strain parameters and facilitating early detection of dysfunction in high‐risk populations. Future research should focus on standardizing strain analysis and integrating artificial intelligence (AI) to optimize clinical applications.

## Graphical abstract

Speckle‐tracking echocardiography enhances stress echocardiography by enabling reproducible strain analysis. This systematic review confirms its diagnostic and prognostic value across coronary, valvular, and heart failure (HF) conditions, supporting its integration for early dysfunction detection, risk stratification, and patient‐tailored care.

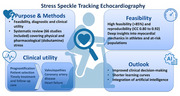

**Abbreviations**
AI: artificial intelligence
CAD: coronary artery disease
CMR‐FT: cardiac magnetic resonance feature tracking
DSE: dobutamine stress echocardiography
GCS: global circumferential strain
GLS: global longitudinal strain
HF: heart failure
ICC: intraclass correlation coefficient
LV: left ventricle/left ventricular
LVAD: left ventricular assist device
LVEF: left ventricular ejection fraction
MeSH: medical subject headings
RV: right ventricle/right ventricular
STE: speckle‐tracking echocardiography
STEMI: ST‐elevation myocardial infarction
VHD: valvular heart disease
WMA: wall motion analysis

## 1 Introduction

Stress echocardiography has become a cornerstone for evaluating suspected coronary artery disease (CAD) and other cardiovascular conditions. It provides critical insights into myocardial function under stress, aiding in the diagnosis of ischemia, valvular disease severity, and myocardial viability. While traditionally reliant on the subjective visual interpretation of wall motion abnormalities, recent advancements such as speckle‐tracking echocardiography (STE), have enhanced its diagnostic capabilities.

STE is a noninvasive imaging technique that quantitatively assesses myocardial deformation through parameters like strain and strain rate, offering reproducible and objective data on myocardial mechanics [1, 2]. This approach is less operator‐dependent and enables early detection of subclinical dysfunction that may not be apparent using traditional methods. This is especially relevant in patients with diabetes mellitus, hypertension, and cardiomyopathies, where early therapy intervention can significantly influence disease progression and outcomes [3, 4, 5]. Moreover, STE enhances the evaluation of left ventricular (LV) function by evaluating myocardial mechanics comprehensively, including longitudinal, circumferential, and radial strain. These detailed insights enable a more accurate assessment of the extent and severity of myocardial ischemia and other cardiac pathologies [1, 2].

Incorporating STE during physical or pharmacological stress testing represents the next logical step in detecting stress‐induced changes in myocardial function and promises greater objectivity and diagnostic precision. However, challenges such as the need for high‐quality imaging, standardization of protocols, and specialized training have limited its widespread adoption. Addressing these barriers, as well as continued research, are essential for maximizing the clinical benefits of STE in stress echocardiography.

This review critically evaluates the feasibility and clinical utility of combining STE with stress echocardiography, examining its methodological rigor, applications in healthy individuals and in at‐risk populations, and its diagnostic potential in CAD and cardiomyopathies. By synthesizing current evidence, we aim to highlight its clinical implications and identify directions for future research.

## 2 Methodology

A comprehensive literature search was conducted to identify studies exploring the use of STE during physical stress testing (e.g., exercise or bicycle stress testing) or pharmacological stress testing with dobutamine. The search encompassed articles published in PubMed up to December 28, 2024. A combination of keywords and Medical Subject Headings (MeSH) terms was utilized to optimize the search strategy. Keywords included “speckle tracking echocardiography,” “stress echocardiography,” “physical stress testing,” “pharmacological stress testing,” “dobutamine,” “myocardial strain,” and “cardiac mechanics.” Corresponding MeSH terms included “Echocardiography, Stress,” “Exercise Test,” “Dobutamine,” and “Myocardial Contraction.” The search string was constructed using Boolean operators to combine the relevant keywords and MeSH terms. The final search string employed was

(“speckle tracking echocardiography” OR “strain analysis”) AND (“stress echocardiography” OR “exercise echocardiography” OR “pharmacological stress testing”)

Studies were included if they investigated STE during physical (exercise or bicycle) or dobutamine stress testing, reported outcomes related to myocardial strain or cardiac mechanics, were published in English, and involved human subjects. Search results from January 1, 2009, to December 28, 2024, were eligible. Exclusion criteria included studies focusing on other imaging modalities or nonhuman subjects. Additionally, studies reporting only data on left atrial strain were excluded. The initial search results were screened for relevance based on titles and abstracts. Full‐text articles were then assessed against the inclusion and exclusion criteria. Data extraction and quality assessment were performed independently by two reviewers, with any discrepancies resolved through consensus.

**FIGURE 1:**
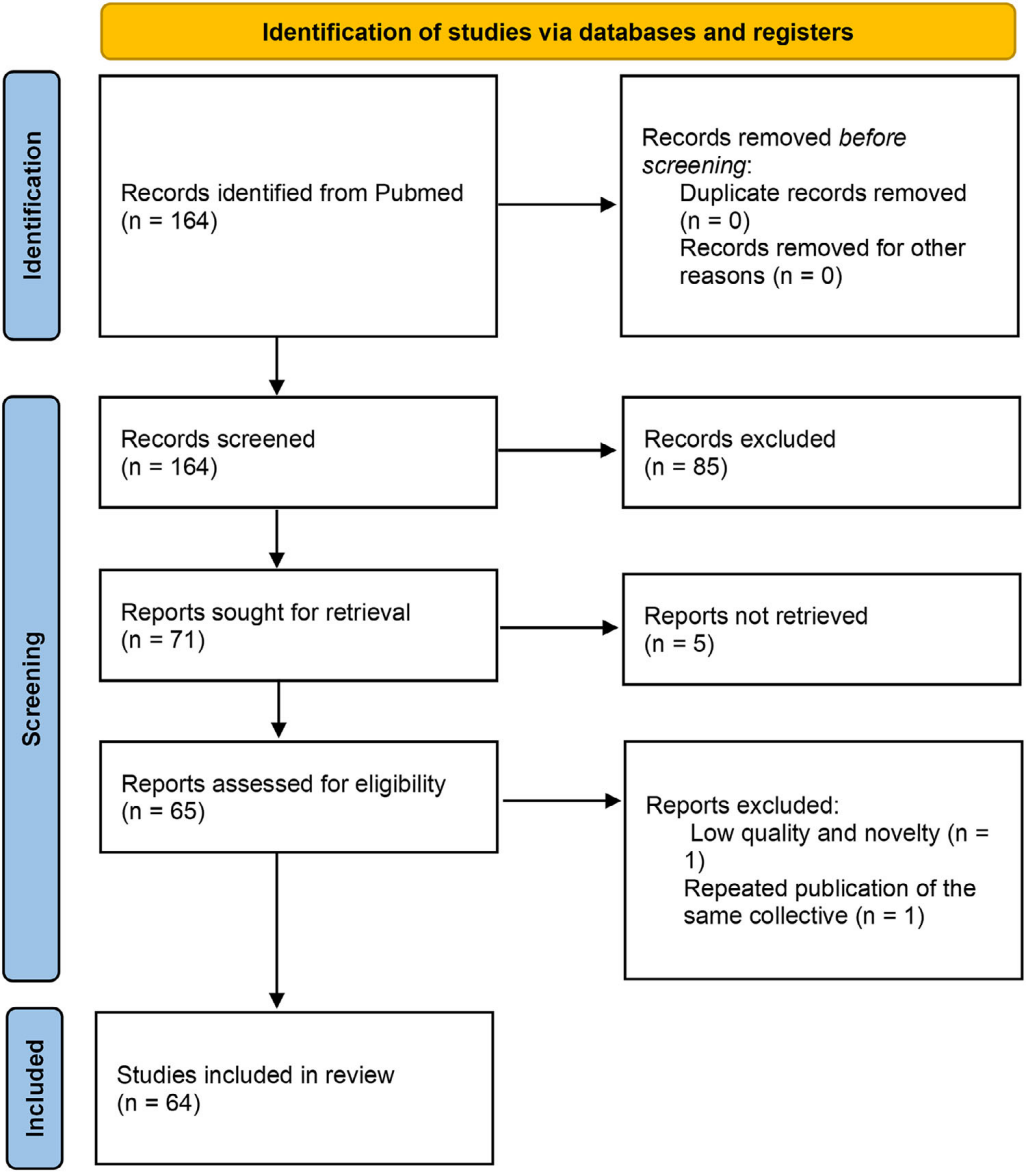
PRISMA flowchart illustrating the selection process of studies included in the systemic review.

Extracted data were synthesized narratively to provide an overview of findings related to STE during stress testing. Quantitative data, such as effect sizes or diagnostic accuracy, were summarized where applicable. Any discrepancies or conflicting results among studies were highlighted and discussed to provide a balanced interpretation. Sixty‐four studies were included into this structured review as shown in the PRISMA flowchart in Figure 1. The studies, including major findings, are listed in Table S1 in the Data Supplement.

### 2.1 Feasibility and Methodological Studies

One of the major challenges associated with using STE during stress echocardiography is its dependence on image quality, which can be compromised by motion artifacts and echogenicity limitations. These issues are exacerbated during physical stress testing, where increased patient movement, higher breathing frequency, and a narrowed ultrasound window can degrade image quality. In contrast, dobutamine stress echocardiography (DSE) mitigates many of these challenges by maintaining a stationary testing environment, resulting in more stable imaging conditions and potentially higher feasibility.

DSE has been shown to provide consistent and reliable STE measurements, particularly in patients with limited echogenicity. In a study by Aboukhoudir et al., only 11.8% of images were affected by poor echogenicity in a cohort of patients with type 2 diabetes, metabolic syndrome, or both, as well as healthy controls [6]. This high feasibility highlights the potential of DSE to facilitate reproducible STE assessments, especially in populations where physical exertion may be contraindicated or difficult. Conversely, physical stress echocardiography often results in higher rates of nonevaluable segments, particularly in the anterior and anteroseptal walls, due to movement artifacts. Von Scheidt et al. reported that up to 15% of myocardial segments were nonevaluable during submaximal exercise stress testing in a young healthy cohort [7]. Despite this limitation, their study demonstrated high interobserver reliability, with intraclass correlation coefficients (ICCs) ranging from 0.80 to 0.92, reinforcing the reproducibility of STE when high‐quality images are obtained. An early study from 2009 from Govind et al. also showed good inter‐ and intraobserver reliabilities in their study about the feasibility of STE in DSE of patients after myocardial infarction [8].

Additional insights into STE feasibility were provided by Wilke et al. and Pieles et al., who evaluated strain and strain rate reproducibility in pediatric patients undergoing ergometric stress testing [9, 10]. Their findings indicated that STE‐derived parameters remained highly reproducible, with image quality showing no significant impact on strain reliability. These results indicate that, despite imaging challenges, STE remains clinically viable when rigorous methodologies are followed.

While most feasibility studies focus on the LV, recent research has highlighted the challenges and potential of STE in assessing right ventricular (RV) strain during stress echocardiography. RV longitudinal strain has gained increasing interest due to its diagnostic and prognostic value in several cardiovascular conditions. However, RV imaging during stress remains technically demanding, primarily due to RV's complex anatomy, thin walls, and frequent interference from adjacent structures.

A study by Sanz‐de la Garza et al. specifically examined the feasibility and reproducibility of RV strain measurements during stress echocardiography, evaluating whether including all six RV segments rather than only the free wall improved assessment reliability [11]. Their findings demonstrated that this approach resulted in significantly higher reproducibility, particularly at higher heart rates, where standard RV free wall strain analysis tended to be less reliable.

Another methodological challenge in stress STE is the impact of high heart rates on software compatibility. Standard echocardiographic systems are optimized for resting heart rates, and during stress echocardiography, heart rates can double or triple, potentially exceeding the software's capability to track myocardial deformation accurately. While none of the reviewed studies explicitly identified this as a major limitation, it remains a potential source of error. Fixsen et al. investigated strain‐based dyssynchrony parameters in heart failure (HF) patients and reported moderate‐to‐good interobserver reliability (ICCs: 0.65 to 0.85), but test–retest reliability was significantly lower (ICCs as low as 0.40 under peak stress) [12]. This suggests that strain values may be affected by heart rate variability and software adaptation, underscoring the need for further research in this area.

Other studies have investigated the correlation between longitudinal strain and visual wall motion assessment during stress echocardiography, showing that STE can enhance the identification of contractility impairment [13, 14]. However, operator expertise remains a critical factor in ensuring accurate interpretation of strain‐based assessments during stress testing.

In summary, DSE offers a more controlled environment for STE, reducing motion‐related artifacts and improving feasibility. Physical stress echocardiography is feasible, but image quality remains a limiting factor, particularly in anterior myocardial segments and RV strain analysis. While segmental strain variability increases under stress, studies such as Wierzbowska‐Drabik et al. suggest that understanding these differences is essential for accurate interpretation [15]. Furthermore, software optimization for high heart rates is needed to enhance strain analysis at peak stress.

Standardized protocols, including adjustments in RV segmentation, consideration of strain heterogeneity, and software improvements, will be essential for ensuring accurate and reproducible STE assessments during stress echocardiography. Addressing these feasibility challenges through technological advancements and optimized imaging strategies will further expand STE's clinical applications.

### 2.2 Healthy Individuals and Exercise Impact Studies

Before evaluating STE in stress echocardiography for pathological conditions, it is essential to establish normal or healthy myocardial strain patterns under physical or pharmacological stress. Traditionally, in visual‐only assessments of stress echocardiography, LV function is expected to remain stable or increase, reflecting contractile reserve and a healthy cardiovascular state. STE provides quantitative insights into myocardial strain, offering a more nuanced understanding of cardiac function during stress.

The study by von Scheidt et al. provides a comprehensive evaluation of myocardial strain in healthy adolescents and young adults during physical stress [7]. This study demonstrated that LV GLS and global circumferential strain (GCS) both increased during submaximal exercise, indicating enhanced myocardial contractility under stress conditions. Specifically, mean LV GLS improved from −20.4% at rest to −23.7% at submaximal stress, while GCS showed a similar pattern of improvement. Additionally, strain rates—reflecting the speed of myocardial deformation—also increased significantly with exercise, supporting the concept of robust contractile reserve in healthy individuals. It is noteworthy that this study used a Philips echocardiography system, and variations in strain values may occur with different manufacturers due to discrepancies in software algorithms and image processing [16]. Similar findings were reported by Cifra et al. and Liu et al., who evaluated myocardial responses in pediatric cohorts using comparable stress protocols [17, 18]. However, greater variability in STE values at higher heart rates suggests the need for age‐specific reference values for clinical use.

Leitman et al. further examined normal myocardial strain responses during stress echocardiography, reinforcing these findings by defining reference values for strain under stress conditions [19]. Their results provide an essential foundation for distinguishing normal physiological responses from early‐stage cardiac dysfunction.

Kleinnibbelink et al. extended this understanding by examining the effects of high‐intensity running on LV and RV function [20]. Their study demonstrated evidence of exercise‐induced cardiac fatigue in both ventricles, with the RV showing greater susceptibility due to higher wall stress during exercise. STE revealed significant reductions in LV and RV strain postexercise, with more pronounced declines observed during low‐to‐moderate stress echocardiography compared to resting conditions. Interestingly, hypoxia, which increases RV afterload, did not exacerbate these reductions, suggesting that factors beyond afterload contribute to exercise‐induced cardiac fatigue in athletes.

Understanding these normal responses is critical for interpreting STE data, particularly in distinguishing physiological adaptations from early pathological changes in athletes. For example, STE can differentiate between beneficial adaptations in well‐trained athletes and potential maladaptive responses indicative of underlying cardiac conditions.

One notable study by Stewart et al. further explored the impact of high‐intensity endurance exercise on myocardial mechanics, illustrating a phenomenon often referred to as “cardiac fatigue” [21]. In this study, competitive cyclists were subjected to a 60‐min high‐intensity cycling challenge, and subsequent echocardiographic assessments revealed a significant reduction in both LV and RV GLS. Specifically, LV GLS decreased by 9.2% immediately postexercise, and RV GLS showed a similar decline. These reductions were exacerbated during subsequent low‐dose physical stress echocardiography, highlighting the cumulative impact of intense exercise on myocardial strain.

A follow‐up study by the same research group, using a similar study protocol, demonstrated that the deterioration in longitudinal strain was not uniform across the myocardium [22]. Instead, certain segments, particularly the septal segments of the LV and the RV free wall, exhibited the most pronounced reductions, likely due to their unique myocardial architecture and fiber orientation. Additionally, these regions are among the first to adapt in the development of the athlete's heart, as they are sensitive to pressure changes and alterations in cardiac index. These findings underscore the need for detailed regional assessments when evaluating exercise‐induced myocardial changes.

Huang et al. investigated the effects of high‐intensity interval training versus moderate‐intensity continuous training on LV mechanics, showing that high‐intensity interval training produced superior improvements in LV contractility and strain response under stress conditions [23]. These findings suggest that training intensity may influence myocardial adaptations, further emphasizing the value of STE in monitoring cardiac function in athletes.

Such studies emphasize the value of STE in evaluating athletes, providing insights into regional myocardial function that can distinguish physiological adaptations from potential maladaptations. This distinction is particularly important for athletes, where subtle differences in strain patterns may have significant clinical implications.

In summary, establishing baseline myocardial strain responses to physical stress is essential for leveraging STE`s full potential in clinical and research contexts. The studies discussed here illustrate STE`s sensitivity in detecting subtle myocardial changes that might be missed by conventional echocardiographic techniques. These insights are particularly valuable for evaluating amateur and professional athletes, some of whom participate in extreme endurance events such as marathons, triathlons, and Ironman races. Addressing the needs of this growing population requires careful differentiation between healthy adaptations and early signs of cardiac pathology, underscoring the importance of ongoing research in this field.

### 2.3 Subclinical Cardiac Dysfunction in At‐Risk Populations

The early identification of patients at high risk for severe cardiac events and the implementation of effective preventive measures represent critical challenges in cardiovascular medicine. STE has demonstrated its predictive utility in resting conditions across various cardiovascular risk factors [3, 24, 25, 26]. However, pathological changes may manifest earlier under stress conditions than at rest, suggesting that stress STE could enhance risk stratification strategies. This hypothesis has been explored in populations with key cardiovascular risk factors: type 2 diabetes mellitus (T2DM), arterial hypertension, and cardiotoxic cancer therapies.

T2DM predisposes to diabetic cardiomyopathy even in the absence of ischemia or hypertension. In patients with T2DM and nonobstructive CAD, Wierzbowska‐Drabik et al. found that LV GLS was lower in diabetic than in nondiabetic patients at every stage of dobutamine stress, indicating an additive strain burden beyond obstructive CAD [27]. Similarly, Harada et al. demonstrated that myocardial deformation was impaired in diabetic individuals before overt systolic dysfunction occurred, particularly in myocardial regions with high metabolic demand [28]. These studies reinforce the importance of early screening with stress STE in diabetic populations.

In arterial hypertension, early detection of myocardial dysfunction prior to the onset of clinical symptoms is critical to prevent progression to HF. Hensel et al. examined 46 adults with well‑controlled essential hypertension and 46 healthy controls using bicycle stress‑STE. Despite normal LVEF, hypertensive subjects showed blunted increases in both circumferential and longitudinal strain and strain‑rate during exercise. These findings highlight reduced myocardial reserve preceding overt dysfunction [29]. Nesti et al. further observed that early hypertensive patients exhibit subtle impairments in myocardial strain under stress, and Cusma Piccione et al. confirmed similar findings in young hypertensive individuals [30, 31]. Roberts et al. reported preserved resting strain but reduced exercise capacity in diabetic–hypertensive patients, suggesting that extra‑cardiac factors (e.g., vascular stiffness) may further limit performance [32].

The cardiotoxic effects of specific chemotherapeutic agents, particularly anthracyclines, are well‐documented. Early detection of subclinical cardiac dysfunction in cancer survivors is essential for the prevention of HF. Khouri et al. evaluated breast cancer survivors treated with doxorubicin‐containing chemotherapy and demonstrated that, despite preserved resting LVEF, approximately 20% exhibited impaired GLS and reduced augmentation of cardiac output during exercise stress echocardiography, which correlated closely with reduced peak oxygen uptake [33]. Von Scheidt et al. examined childhood cancer survivors using STE during submaximal exercise [34]. While resting LV strain values were comparable between cancer survivors and healthy controls, progressive reductions in strain were observed during exercise in the cancer survivor group, indicative of impaired myocardial adaptation to stress. Similarly, Yazaki et al. found that patients who underwent anthracycline chemotherapy exhibited impaired contractile reserve during stress conditions, even when resting strain values were within normal limits [35]. These findings emphasize the value of stress STE as a screening tool for cardiotoxicity in cancer survivors and individuals undergoing chemotherapy.

The application of STE during stress echocardiography provides unique insights into early subclinical cardiac dysfunction across diverse at‐risk populations. By identifying subtle changes in myocardial mechanics under stress, stress STE enables earlier intervention, potentially mitigating the progression to overt HF. The integration of stress STE into routine evaluations could enhance risk stratification and inform targeted preventive strategies for individuals at risk of cardiovascular events. Further research is needed to standardize stress STE protocols across different patient populations and imaging vendors, ensuring broad clinical applicability and diagnostic reliability

### 2.4 Valvular heart disease (VHD)

VHDs encompass a range of pathologies affecting the heart's valve structures, leading to significant morbidity and mortality worldwide. Accurate assessment of these conditions is crucial for determining the appropriate timing of interventions and for prognostic evaluation. While traditional echocardiographic techniques provide essential anatomical and functional information, they may not fully capture subtle myocardial dysfunction associated with VHDs. Stress echocardiography, particularly when combined with STE, offers enhanced diagnostic and prognostic capabilities by evaluating myocardial deformation under stress conditions [36, 37].

Progressive volume overload can mask early systolic impairment at rest. In asymptomatic severe MR, Neveu et al. showed that failure of GLS and myocardial work to rise during bicycle stress was an independent predictor of adverse events [38]. Žvirblytė et al. further demonstrated that patients who preserved LV contractile reserve during exercise also maintained better RV strain, highlighting the biventricular nature of MR adaptation [39].

Low‐dose dobutamine stress combined with STE refines timing of aortic valve replacement (AVR). In 50 patients with severe AR and markedly reduced LVEF, Li et al. found that baseline GLS and, more powerfully, peak‐stress GLS (cut‐off −9.4%) independently predicted postoperative recovery of LVEF; an absolute GLS improvement ≥ 1.9% delineated responders [40]. Earlier resting‐only studies had already linked depressed GLS to poor outcome [41]; the Li data confirm that contractile reserve, not the resting value, is the decisive metric when LVEF is low.

Contractile reserve differs fundamentally between pressure‐ and volume‐load hypertrophy. In a matched study of patients with concentric LVH caused by asymptomatic moderate‐to‐severe aortic stenosis (AS) versus hypertrophic cardiomyopathy (HCM), Schnell et al. showed that during submaximal cycling, GLS fell in AS (Δ +0.9 ± 3.1%), whereas it rose in HCM (Δ −1.9 ± 3.2%; *p* = 0.003) [42]. The divergent behavior, despite identical wall thickness at rest, underscores the dominant influence of afterload on longitudinal fibers and illustrates how stress‐STE can discriminate etiology and unmask limited reserve in AS long before symptoms occur.

While current data on stress STE in VHD remain limited, these findings suggest that its integration into routine valvular assessment could provide valuable prognostic insights. Future studies should focus on standardizing strain thresholds and evaluating their role in guiding clinical decision‐making in larger patient cohorts.

### 2.5 CAD and Myocardial Ischemia

**FIGURE 2:**
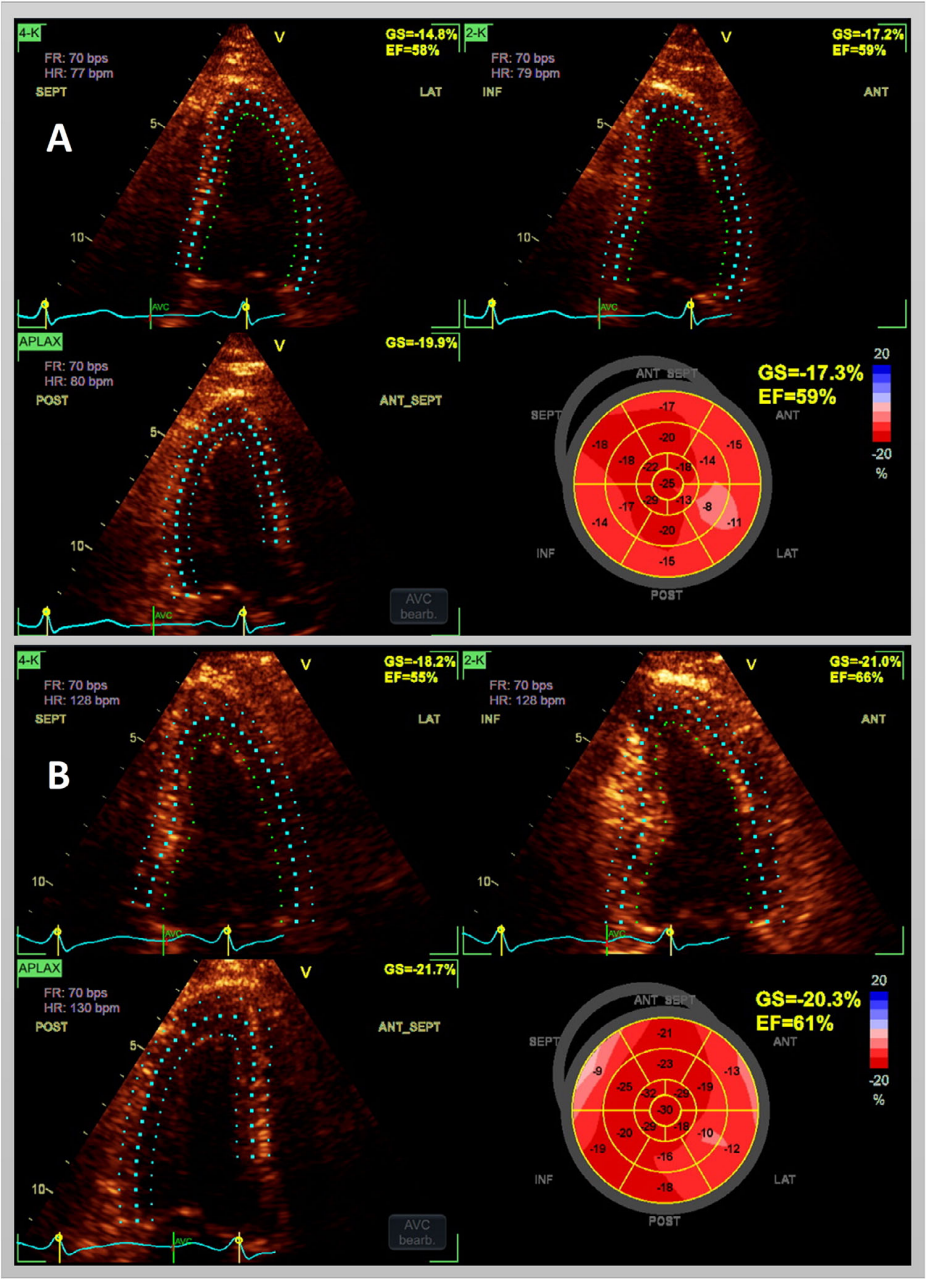
A 69‐year‐old female patient with a history of PCI of the left anterior descending artery following an acute coronary syndrome. The figure demonstrates stress echocardiography performed on a S70N system (GE Vingmed Ultrasound AS, Horten, Norway) with two‐dimensional left ventricular longitudinal strain imaging. At rest (A), there is a reduction in regional longitudinal strain in the anterior wall, which is more pronounced in the anterolateral wall. Under stress, using a semisupine bicycle test at maximum exertion (100 watts) (B), global and regional longitudinal strain improves, indicating good contractile reserve and no new signs of ischemia. PCI, percutaneous coronary intervention.

Stress echocardiography is a well‐established diagnostic tool for CAD and myocardial ischemia, providing essential insights into myocardial perfusion and wall motion abnormalities under stress conditions. However, its accuracy depends heavily on operator expertise, introducing subjectivity and interobserver variability. STE has been proposed to enhance diagnostic precision by providing quantifiable strain parameters that are less operator‐dependent. While several studies support its integration into stress echocardiography, others emphasize the continued value of conventional wall motion analysis (WMA), particularly in experienced hands. An example of a stress STE examination of a patient with CAD is shown in Figure 2.

Several studies have demonstrated that STE improves ischemia detection, particularly when used alongside WMA. A multicenter study by Ng et al. found that LV GLS during DSE achieved diagnostic accuracy comparable to WMA, with improved sensitivity when both methods were combined [43]. Nagy et al. similarly observed in a retrospective analysis of contrast‐enhanced DSE that adding strain analysis to WMA improved diagnostic performance, particularly in detecting functionally significant CAD [44].

In post‐STEMI patients, Joyce et al. identified a > 1.9% reduction in peak systolic longitudinal strain as a sensitive marker of residual ischemia, demonstrating a sensitivity of 87% [45]. Elamragy et al. further confirmed that GLS at peak stress improved diagnostic accuracy, achieving a sensitivity of 89.8% and specificity of 84.6%, which outperformed conventional WMA alone [46].

In two multicenter prospective studies, Ilardi et al. (*n* = 88) and Lin et al. (*n* = 89) confirmed that GLS and myocardial work indices during DSE and exercise SE, respectively, improved ischemia identification, particularly in cases of LAD stenosis [47, 48]. Other studies found similar results using DSE and STE, showing enhanced diagnostic accuracies, especially in low or intermediate dobutamine doses and focusing on subendocardial layers [49, 50, 51, 52, 53]. These findings are further supported by Nishi et al. and Farag et al., who independently demonstrated that peak GLS reductions during stress correlated with the severity of CAD assessed by fractional flow reserve measurement (Nishi) or SYNTAX score (Farag), making STE particularly useful for patients with multivessel disease [54, 55]. Rumbnaite et al. prospectively enrolled 145 patients, confirming that STE plus WMA significantly improved CAD detection, particularly in patients with complex coronary anatomy and borderline functional test results [56]. Additionally, Takagi et al. demonstrated that prolonged regional systolic dysfunction following stress testing correlated strongly with ischemic burden, suggesting that STE can detect ischemia beyond the immediate stress phase [57].

Not only detection but also exclusion of CAD is of importance in daily practice. Karlsen et al. showed that normal exercise‐induced GLS effectively ruled out significant CAD with a high negative predictive value and an AUC of 0.97 [58]. Accordingly, Mansour et al. reported that a peak exercise GLS ≥ 20% effectively excluded obstructive CAD compared with coronary CT angiography [59]. On the other hand, this trial showed inferior diagnostic accuracy of peak GLS in ruling in CAD compared to WMA. Also challenging a potential superiority of STE over WMA was a study by Uusitalo et al. They directly compared STE‐derived strain indices with WMA and found that, while strain parameters correlated with ischemia, their diagnostic accuracy was not superior to expert visual analysis [60]. Further reinforcing this, Wierzbowska‐Drabik et al. reported that automated GLS measurements showed good agreement but did not outperform conventional visual assessments in ischemia detection [61].

Some studies suggest that STE's strength lies in refining ischemia localization rather than replacing WMA entirely. Shenouda et al. demonstrated that regional strain rate analysis was more effective than global strain in identifying ischemic segments, particularly in patients with prior revascularization [62].

Beyond its diagnostic role, STE has demonstrated significant prognostic value. Bansal et al. reported that stress‐induced GLS reductions were associated with long‐term cardiovascular events, reinforcing STE's predictive value beyond ischemia detection [63]. Liu et al. found that combining low‐dose DSE with STE significantly improved sensitivity and specificity for detecting coronary microvascular obstruction, achieving accuracy comparable to cardiac MRI feature tracking [64]. Also, viability testing was enhanced by adding STE to conventional low‐dose DSE as shown in two separate studies with patients after myocardial infarction [65, 66].

The integration of artificial intelligence (AI) into STE analysis has also emerged as a promising avenue. The PROTEUS trial, presented at ESC 2024, highlighted that AI‐enhanced strain imaging can reduce interobserver variability, improve diagnostic efficiency, and provide more standardized ischemia detection [67].

While STE offers quantifiable and reproducible strain parameters that enhance ischemia detection, it does not consistently outperform expert visual WMA in all scenarios. Current evidence suggests that STE should complement, rather than replace, conventional WMA, particularly in centers with high operator experience. The combined approach of STE and WMA appears to offer the highest diagnostic yield, with potential future advancements in AI further improving standardization and clinical applicability. Further studies are needed to define optimal STE thresholds, ensuring its integration where it adds the most clinical value.

### 2.6 Cardiomyopathies and HF

HF remains a major challenge in cardiology, contributing significantly to morbidity, mortality, and healthcare burden. Accurate prognostic assessment is crucial in HF management, guiding decisions about advanced interventions such as implantable cardioverter defibrillators or cardiac resynchronization therapy. While traditional measures like LVEF are widely used, they often fail to capture the nuanced myocardial mechanics and contractile reserve necessary for accurate risk stratification. STE provides detailed insights into global and segmental myocardial strain, offering valuable prognostic information for both left and RV function.

Exercise intolerance, a hallmark of HF, is poorly correlated with conventional resting echocardiographic parameters such as LVEF. Tsougos et al. demonstrated that in patients with HF with reduced ejection fraction, GLS during peak exercise correlated significantly better with exercise tolerance than LVEF itself [68]. Similarly, Mizukoshi et al. emphasized that in HCM, the early diastolic strain rate measured during stress echocardiography was more closely associated with exercise capacity than systolic function alone [69]. Tan et al. further revealed that patients with HF with preserved ejection fraction demonstrate complex abnormalities of both systolic and diastolic function during exercise—including impaired longitudinal shortening, reduced torsion, and delayed untwisting—which contribute to impaired ventricular suction and diastolic filling [70]. Complementing these findings, Henein et al. showed that systolic and diastolic strain measurements at rest, and more pronounced under stress, correlated well with exercise tolerance in HF patients with preserved ejection fraction, whereas conventional measurements of diastolic function and LV filling pressures such as E/e’ did not [71]. Together, these findings underscore the critical role of stress echocardiography and strain imaging in revealing subclinical dysfunction and predicting exercise capacity in HF.

Mitro et al. evaluated patients undergoing cardiac resynchronization therapy using DSE. They found that patients with contractile reserve in the myocardial region targeted by the LV lead were significantly more likely to respond to therapy [72].

The role of RV function in HF has been historically underemphasized, despite its recognized prognostic importance. At rest, RV dysfunction is a well‐established predictor of HF progression and increased mortality, highlighting the importance of stress‐induced RV function assessments [73]. D'Andrea et al. examined the role of stress STE in patients with early‐stage idiopathic pulmonary fibrosis. They observed that a reduction in RV longitudinal strain during stress was already present at an early stage of the disease and correlated with hemodynamic and clinical markers suggesting a role in early prognostication [74]. The research group of Matsumoto et al. investigated the prognostic value of contractile reserve during dobutamine stress in patients with dilated cardiomyopathy using 3D GLS and GCS as well as RV strain. They demonstrated that a lack of improvement—or even a deterioration—in global LV GCS and especially RV free wall strain under dobutamine infusion was associated with a significantly increased risk of adverse cardiovascular events [75, 76].

Stapór et al. investigated RV deformation in patients with left ventricular assist devices (LVADs), assessed on average 7.3 months postimplantation [77]. They found that RV strain parameters did not deteriorate after LVAD implantation. Under exercise conditions, neither RV GLS nor RV free wall strain changed significantly, indicating the absence of RV contractile reserve. These findings suggest that exercise stress strain imaging may offer valuable prognostic insights and could support optimization of post‐LVAD management.

The application of STE in HF offers unparalleled insights into myocardial mechanics, providing both functional and prognostic data that surpass traditional metrics like LVEF. While global strain during stress can predict exercise capacity, segmental, and RV strain analysis provide clinically actionable information for risk stratification and management.

Emerging AI models are being trained to automate STE analysis, standardize risk prediction, and reduce interobserver variability. Future studies will determine whether these technologies can integrate into clinical workflows to enhance HF diagnostics and decision‐making.

## 3 Conclusion

STE has emerged as a valuable tool in stress echocardiography, providing quantifiable and reproducible strain parameters that enhance the assessment of myocardial function. This systematic review confirms that STE integrated into stress echocardiography is feasible, improves ischemia detection, risk stratification. However, broader clinical adoption remains challenged by methodological issues that warrant further attention.

Among these, image quality limitations during physical stress, reduced tracking accuracy at high heart rates, and especially anatomical complexities such as the thin‐walled right ventricle constrain measurement consistency. Furthermore, variability between different vendors’ software packages continues to limit cross‐study comparability and clinical standardization. Recognizing this, international imaging societies are actively working toward consensus‐based protocols and vendor‐neutral solutions.

AI‐assisted STE interpretation represents a promising avenue for improving diagnostic accuracy and reducing interobserver variability. However, prospective studies are required to validate AI‐driven strain algorithms in real‐world clinical settings. Additionally, further investigations should explore the prognostic value of STE in large, multicenter cohorts, particularly in patients with borderline CAD, cardiomyopathies, and VHD.

Finally, while current evidence supports the complementary role of STE alongside WMA, its ability to replace conventional ischemia assessment methods remains uncertain. Future trials should evaluate whether strain‐based approaches improve clinical decision‐making and patient outcomes, particularly in cases where traditional WMA provides inconclusive results. Addressing these gaps will be crucial in fully integrating STE into routine stress echocardiography practice.

## Conflicts of Interest

The authors declare no conflicts of interest.

## Supporting information




**Supporting Table 1**: Summary of Included Studies in Systematic Review.
