# Supplementary material for: Applications of Speckle Tracking Echocardiography in Stress Echocardiography: A Systematic Review on Feasibility, Diagnostic, and Clinical Utility
Source: Echocardiography. 2025 Aug 6;42(8):e70251. doi: 10.1111/echo.70251 (PMC12352479; doi:10.1111/echo.70251)
Supplement: Supplementary file 1 — Supporting Table 1: Summary of Included Studies in Systematic Review. [file ECHO-42-e70251-s001.docx]

# Table S1. Summary of Included Studies in Systematic Review

| **Study (Author, Year)** | **Population** | **Stress Modality** | **STE Parameters Analyzed** | **Key Findings** | **Clinical Relevance** |
| --- | --- | --- | --- | --- | --- |
| **Feasibility and Methodological Studies** | | | | | |
| Aboukhoudir et al., 2022 | T2DM, MetS patients + controls  (n=158) | DSE | GLS, GCS | Low percentage of poor image quality, high reproducibility | Demonstrates DSE feasibility even in T2DM |
| von Scheidt et al., 2020 | Healthy adolescents (n=50) | Exercise SE | GLS, GCS, strain rate | Feasible with limitations in anterior segments | Establishes reference strain values during stress |
| Wilke et al., 2018 | Pediatric patients (n=127) | Exercise SE | GLS, strain rate | High reproducibility; image quality did not significantly affect measurements | Supports STE feasibility in children under stress |
| Govind et al., 2009 | Patients after acute myocardial infarction (n=29) | DSE | LV strain and strain rates | Feasible with good inter- and intraobserver reliability | Deeper insights in myocardial mechanics patients with myocardial infarction |
| Pieles et al., 2015 | Adolescents (n=23) | Exercise SE | Biventricular strain | Strain reproducible across incremental exercise stages | Validates biventricular strain assessment in youth during exercise |
| Sanz-de la Garza et al., 2019 | Healthy adults (n=80) | Exercise SE | RV strain | Using all RV segments improved reproducibility, especially at high HR | Optimizes RV assessment protocol under stress |
| Fixsen et al., 2022 | HFrEF patients (n=18) | Exercise SE | Dyssynchrony parameters (strain) | Good interobserver reliability, but lower test-retest at peak stress | Indicates need for software optimization under high HR conditions |
| Wierzbowska-Drabik et al., 2017 | Patients with chest pain undergoing DSE (n = 238) | DSE | GLS, strain rate | Stress induced impairment in strain and strain rate detected visual assessed wall motion abnormalities | Quantitative strain analysis complements visual wall‑motion to identify inducible contractility impairment during DSE |
| Wierzbowska-Drabik et al., 2017 | High CAD risk (non-stenosed segments) (n=111) | DSE | Segmental longitudinal strain | Strain variability exists in non-stenosed segments; interpretation requires caution | Underlines strain heterogeneity even in non-ischemic segments |
| **Healthy Individuals and Exercise Impact Studies** | | | | | |
| Cifra et al., 2016 | Healthy children (n=62) | Exercise SE | Systolic and diastolic strain | Exercise increases strain and SR; confirms contractile reserve | Provides pediatric normative strain response data |
| Kleinnibbelink et al., 2021 | Trained athletes (n=21) | Exercise SE | LV and RV strain | Exercise fatigue with RV predominance; hypoxia had no additive effect | Reveals strain patterns under high-intensity training |
| Liu et al., 2016 | Children (n=97) | Exercise SE | Global strain parameters | Strain increases under supine exercise; reproducible | Demonstrates feasibility and adaptation in pediatric group |
| Leitman et al., 2017 | Healthy adults (n=46) | Exercise SE | GLS | Defines normal GLS responses under stress | Establishes reference strain values for adult stress echocardiography |
| Stewart et al., 2015 | Endurance cyclists (n=15) | Exercise SE | LV GLS, RV GLS | Post-exercise reduction in strain (cardiac fatigue) | Identifies physiological strain response to exercise |
| Stewart et al., 2017 | Endurance athletes (n=23) | Exercise SE | Regional LV, RV strain | Greatest strain reduction in septal and RV free wall | Shows regional cardiac fatigue patterns in athletes |
| Huang et al., 2019 | Healthy young adults (n=54) | Exercise SE | GLS | High-intensity interval training leads to greater strain improvements | Supports training-specific myocardial adaptation |
| **Subclinical Cardiac Dysfunction in At-Risk Populations** | | | | | |
| Hensel et al., 2014 | Hypertensive adults and healthy controls (n=84) | Exercise SE | LV strain and strain rate | Reduced LV strain & SR at rest, exaggerated during exercise despite normal EF | Exercise-STE detects subclinical LV systolic dysfunction for earlier risk stratification |
| Wierzbowska-Drabik et al., 2017 | CAD and T2DM patients  (n=127) | DSE | GLS | Reduced GLS in T2DM across all stress stages | Shows additional strain burden in diabetic heart beyond coronary artery disease |
| Harada et al., 2024 | Post-COVID children and controls (n=55) | Exercise SE | GLS | Impaired reserve despite normal rest function | Demonstrates residual subclinical myocardial impact in pediatric post-COVID cohort |
| Von Scheidt et al., 2022 | Childhood cancer survivors and controls (n=127) | Exercise SE | GLS, GCS, strain rate | Prevalence of abnormal GLS rose with stress (1.3 % rest → 8.6 % submax); GCS increase was lower in cancer survivors | Stress‑strain reveals subclinical LV adaptation deficits in survivors; aids early cardio‑oncology surveillance |
| Khouri et al., 2014 | Breast cancer patients and controls (n=77) | Exercise SE | GLS | GLS reduction linked with microvascular dysfunction post-chemo | Detects early cardiotoxicity before EF changes |
| Roberts et al., 2020 | T1DM, T2DM patients and controls (n=51) | Exercise SE | GLS, twist | Normal strain at rest; reduced exercise capacity unrelated to twist or strain | Points to vascular limitations in diabetic patients |
| Nesti et al., 2021 | T2DM patients (n=88) | Exercise SE | GLS | Subtle strain impairment during stress despite normal rest function | Identifies early diabetic myocardial dysfunction |
| Cusma Piccione et al., 2017 | Young hypertensive patients and controls (n=54) | Exercise SE | Strain reserve | Reduced reserve under stress vs. normotensives | Reveals maladaptation in early-stage hypertension |
| Yazaki et al., 2020 | Repaired TOF patients (n=13) | Exercise SE | Layer-specific strain | Impaired regional contractile reserve post-repair | Early detection of functional deterioration post-congenital repair |
| **Valvular Heart Disease** | | | | | |
| Neveu et al., 2024 | Asymptomatic severe MR patients (n=103) | Exercise SE | GLS and myocardial work | Reduced strain reserve predicted adverse outcomes | Helps optimize timing of surgery in primary MR |
| Žvirblytė et al., 2020 | Moderate-severe MR patients (n=51) | Exercise SE | LV and RV GLS | RV function did not differ from controls; patients with preserved LV contractile reserve showed better RV deformation | LV contractile reserve modulates RV response to stress in asymptomatic MR |
| Schnell et al., 2013 | AS (n = 25) vs HCM (n = 25), matched concentric LVH | Exercise SE | GLS, GCS | GLS ↓ in AS but ↑ in HCM; ΔGLS correlated with afterload | Differentiates pressure‑ vs volume‑induced LVH; identifies limited reserve in asymptomatic AS |
| Li et al., 2023 | Patients with severe AR and HFrEF (n = 50) | Low‑dose DSE | Baseline & peak GLS | Peak‑stress GLS > –9.4 % predicted postoperative LVEF > 40 % (AUC 0.895) | Guides timing of AVR in severe AR with low EF |
| **Coronary Artery Disease and Myocardial Ischemia** | | | | | |
| Ng et al., 2009 | Patients with suspected CAD (n=103) | DSE | LV GLS | GLS improved sensitivity of ischemia detection when added to WMA | Increased diagnostic accuracy for CAD with STE + WMA |
| Nagy et al., 2015 | Patients with suspected CAD (n=60) | DSE | LV GLS | Strain analysis added diagnostic value to contrast-enhanced WMA | Combining strain and contrast improves stress echo performance |
| Joyce et al., 2015 | Post-STEMI patients (n=105) | DSE | Longitudinal strain | 1.9% peak strain reduction identified residual ischemia | Sensitive marker for residual ischemia post-MI |
| Elamragy et al., 2020 | Patients with moderate CAD probability (n=101) | DSE | Peak GLS | GLS had 89.8% sensitivity, 84.6% specificity | Superior diagnostic yield over visual analysis alone |
| Ilardi et al., 2021 | Suspected CAD (n=88) | DSE | Global and regional strain | Peak GLS accurately detected LAD stenosis | Highlights regional utility of strain analysis |
| Lin et al., 2021 | Suspected CAD (n=89) | Exercise SE | Layer strain, myocardial work | Improved accuracy with combined GWE and strain | Supports advanced treadmill-based diagnostics |
| Liu et al., 2022 | Post-PCI STEMI patients (n=61) | Low‑dose DSE | GLS | Comparable diagnostic value to CMR-FT | STE effective in post-intervention CAD evaluation |
| Takagi et al., 2011 | Exercise-induced ischemia (n=30) | Exercise SE | Regional strain | Persistent dysfunction post-exercise indicated ischemia | Captures ischemic burden beyond stress phase |
| Nishi et al., 2019 | Suspected CAD (n=30) | Exercise SE | Layer-specific strain | Peak strain reductions correlated with CAD extent | Useful for multivessel disease identification |
| Cadeddu et al., 2019 | Suspected CAD (n=20) | DSE | Subendocardial strain | Subendocardial strain under dobutamine increseases specificity especially at low dobutamine doses | Stress STE enhances accuracy detecting CAD |
| Yu et al., 2013 | Suspected CAD (n=76) | DSE | Strain, dyssynchrony | Detected dysfunction at intermediate DSE doses | Early ischemia identification before wall motion changes |
| Yang et al., 2016 | Suspected CAD (n=37) | DSE | GLS, regional strain | Strain map added no benefit over visual assessment; specificity lower for less‑experienced readers | Highlights importance of operator expertise; strain bulls‑eye alone may not improve CAD detection |
| Karlsen et al., 2022 | Unstable angina (n=78) | Exercise SE | GLS | Normal stress GLS ruled out significant CAD | Supports STE for safe exclusion of CAD |
| Uusitalo et al., 2016 | Suspected CAD (n=50) | DSE | GLS, strain rate | STE did not outperform expert WMA | STE complementary but not superior |
| Wierzbowska-Drabik et al., 2014 | High-risk CAD patients without obstruction (n=111) | DSE | AFI vs 2D STE | Good agreement between methods | Supports automated strain analysis feasibility |
| Park et al., 2016 | Suspected CAD (n=121) | DSE | Layer-specific strain | Improved detection accuracy for CAD | Layer-based assessment yields better sensitivity |
| Shenouda et al., 2023 | Patients after PCI or CABG (n=33) | SE | Strain rate | Strain rate was best predictor of CAD | Prior ACS – stress STE stratifies residual risk |
| Li et al., 2016 | Patients with old anterior MI and low LVEF (n=33) | Low‑dose DSE | GLS | STE was comparable to wall-motion analysis detecting viable myocardium | Viability detection using low-dose stress strain |
| Gong et al., 2021 | Patients with prior myocardial infarction (n=42) | Low‑dose DSE | RV free wall strain | Viability detection via stress STE showed better specificity and sensitivity than SPECT-imaging | STE viable alternative to nuclear imaging |
| d'Entremont et al., 2021 | Suspected CAD post-treadmill (n=201) | Exercise SE | GLS | Feasible and accurate for stenosis detection | Validated post-treadmill application of STE |
| Farag et al., 2020 | Stable angina (n=92) | DSE | GLS | Strain predicted angiographic CAD severity | Predictive for significant stenoses |
| Bansal et al., 2010 | Old MI (n=61) | DSE | Strain via TDI and STE | Viability detected accurately with strain analysis | Strain confirms tissue viability |
| Mansour et al., 2018 | Suspected obstructive CAD (n=103) | Exercise SE | GLS at rest/peak | GLS at peak stress >20% showed good results ruling out CAD, while WMA was superior in diagnosing CAD | Supports usage of strain to rule out CAD |
| Rumbinaite et al., 2016 | Moderate-high CAD risk (n=127) | DSE | GLS, diastolic strain rate | Peak‑dose GLS highly sensitive & specific (AUC 0.955); GLS + WMA AUC 0.977 | Supports GLS as primary parameter for CAD detection during DSE |
| Rumbinaite et al., 2022 | Suspected CAD (n=145) | DSE | GLS, regional longitudinal SR, GRS | Confirmed diagnostic utility for significant CAD | Combined STE & WMA approach enhances detection of functionally significant CAD |
| **Cardiomyopathies and Heart Failure** | | | | | |
| Tsougos et al., 2018 | Patients with HFrEF  (n=61) | Exercise SE | GLS | GLS at stress correlates better with exercise tollerance than EF | Supports GLS as marker of exercise limitation |
| Mizukoshi et al., 2013 | HCM patients (n=57) | Exercise SE | Early diastolic strain rate | Diastolic dysfunction during stress correlated with limited exercise capacity | Highlights non-systolic contributors to functional impairment in HCM |
| D’Andrea et al., 2019 | Patients with early idiopathic pulmonary fibrosis and controlls (n=100) | Exercise SE | RV GLS | RV GLS was reduced already at rest and more pronounced at stress and correlated with exertion capacity and pulmonary arterial pressure | RV GLS at rest and stress is a tool for early prognostification in pulmonary fibrosis patients |
| Mitro et al., 2014 | Patients with HFrEF and resynchronisation therapy (n=41) | DSE | Segmental strain | Contractile reserve (increase of strain) in the segment of LV electrodes correlated with response to CRT | Viability confirmed by stress STE may improve patient selection for resynchronization therapy |
| Tan et al., 2009 | HFpEF patients (n=49) | Exercise SE | GLS, torsion, untwisting | Combined systolic and diastolic dysfunction during exertion | Comprehensive functional assessment beyond EF in HFpEF |
| Matsumoto et al., 2012 | DCM patients (n=65) | 3D DSE | GCS | GCS change <2.71% predicted CV events with 83% sensitivity and 80% sesnitivity | Supports prognostic value of 3D strain parameters in DCM patients |
| Matsumoto et al., 2015 | DCM patients (n=104) | 3D DSE | Biventricular strain | Biventricular reserve predicted long-term prognosis | Advanced multidimensional strain for HF risk stratification |
| Stapór et al., 2024 | LVAD patients (n=22) | Post-LVAD follow-up | RV strain | RV worsening post-LVAD; no change of RV strain under exercise (no contractile reserve) | Supports post-implant RV monitoring for prognosis |
| Henein et al., 2022 | HFpEF patients (n=54) | Exercise SE | GLS | LV strain and strain rate as well as LA volume under stress were predictive of functional capacity but not E/e’ | Insights into myocardial mechanics in HFpEF |
